# Rifaximin potentiates clarithromycin against *Mycobacterium abscessus in vitro* and in zebrafish

**DOI:** 10.1093/jacamr/dlad052

**Published:** 2023-05-08

**Authors:** Boon Chong Goh, Simon Larsson, Linh Chi Dam, Yan Han Sharon Ling, Wei Lin Patrina Chua, R Abirami, Samsher Singh, Jun Long Ernest Ong, Jeanette W P Teo, Peiying Ho, Philip W Ingham, Kevin Pethe, Peter C Dedon

**Affiliations:** Antimicrobial Resistance Interdisciplinary Research Group, Singapore-MIT Alliance for Research and Technology Centre, Singapore, Singapore; Lee Kong Chian School of Medicine, Nanyang Technological University, Singapore, Singapore; Antimicrobial Resistance Interdisciplinary Research Group, Singapore-MIT Alliance for Research and Technology Centre, Singapore, Singapore; Antimicrobial Resistance Interdisciplinary Research Group, Singapore-MIT Alliance for Research and Technology Centre, Singapore, Singapore; Antimicrobial Resistance Interdisciplinary Research Group, Singapore-MIT Alliance for Research and Technology Centre, Singapore, Singapore; Antimicrobial Resistance Interdisciplinary Research Group, Singapore-MIT Alliance for Research and Technology Centre, Singapore, Singapore; Lee Kong Chian School of Medicine, Nanyang Technological University, Singapore, Singapore; Lee Kong Chian School of Medicine, Nanyang Technological University, Singapore, Singapore; Department of Laboratory Medicine, National University Hospital, Singapore, Singapore; Antimicrobial Resistance Interdisciplinary Research Group, Singapore-MIT Alliance for Research and Technology Centre, Singapore, Singapore; Lee Kong Chian School of Medicine, Nanyang Technological University, Singapore, Singapore; Institute of Molecular and Cell Biology, Agency of Science, Technology and Research (A*Star), Singapore, Singapore; Lee Kong Chian School of Medicine, Nanyang Technological University, Singapore, Singapore; Singapore Centre for Environmental Life Sciences Engineering, Nanyang Technological University, Singapore, Singapore; Antimicrobial Resistance Interdisciplinary Research Group, Singapore-MIT Alliance for Research and Technology Centre, Singapore, Singapore; Department of Biological Engineering, Massachusetts Institute of Technology, Cambridge, MA, USA

## Abstract

**Background:**

*Mycobacterium abscessus* is a non-tuberculous mycobacterium (NTM) that causes chronic pulmonary infections. Because of its extensive innate resistance to numerous antibiotics, treatment options are limited, often resulting in poor clinical outcomes. Current treatment regimens usually involve a combination of antibiotics, with clarithromycin being the cornerstone of NTM treatments.

**Objectives:**

To identify drug candidates that exhibit synergistic activity with clarithromycin against *M. abscessus*.

**Methods:**

We performed cell-based phenotypic screening of a compound library against *M. abscessus* induced to become resistant to clarithromycin. Furthermore, we evaluated the toxicity and efficacy of the top compound in a zebrafish embryo infection model.

**Results:**

The screen revealed rifaximin as a clarithromycin potentiator. The combination of rifaximin and clarithromycin was synergistic and bactericidal *in vitro* and potent in the zebrafish model.

**Conclusions:**

The data indicate that the rifaximin/clarithromycin combination is promising to effectively treat pulmonary NTM infections.

## Introduction

Infections caused by non-tuberculous mycobacteria (NTM) are recognized as being a fast-rising health threat globally, especially in the context of intractable pulmonary infections.^1^ Among NTMs, the *Mycobacterium avium* and *Mycobacterium abscessus* complexes are the most commonly implicated in human infections.^2,3^*M. abscessus* causes pulmonary infections in patients with immune deficiencies or with underlying lung conditions, such as cystic fibrosis, bronchiectasis or chronic obstructive pulmonary disease.^2,4^ Further, demonstrated transmission of *M. abscessus* between cystic fibrosis patients has increased the urgency to identify novel treatments for this pathogen.^5^ In addition to lung diseases, *M. abscessus* has been implicated in severe infections in cutaneous, joint, soft tissue and surgical sites.^6^ However, chemotherapeutic options for treating *M. abscessus* infections are limited because the bacterium is intrinsically resistant to most antibiotics.^7^

*M. abscessus* infections are treated by a multidrug regimen consisting of clarithromycin, amikacin and either cefoxitin, imipenem or tigecycline.^8,9^ Such treatments are, however, complicated by the ability of some *M. abscessus* subspecies to acquire phenotypic resistance to clarithromycin upon repeated exposure to the drug.^10–12^ The presence of the *erm*(41) gene is responsible for inducible resistance to macrolides.^11^ Because clarithromycin is currently the only highly effective oral antibiotic,^13^ resistance to clarithromycin is of great concern.

Given the importance of clarithromycin, two Phase II/III clinical trials are exploring the potency of clarithromycin-containing drug regimens (clinicaltrials.gov identifier: NCT04630145 and NCT04310930), whereas at the preclinical stage several drug screening campaigns to identify clarithromycin potentiators have been performed. These efforts have produced several drug candidates, such as rifabutin,^14^ omadacycline,^15^ vancomycin,^16^ moxifloxacin^17^ and imipenem.^18^

Here, we performed a whole-cell phenotypic screen using *M. abscessus* with its Erm(41)-mediated macrolide resistance preinduced. This set-up enabled compound screening in combination with clarithromycin at a concentration that would otherwise inhibit the growth of uninduced cells. Therefore, the hits obtained from this screen are more likely to synergize with clarithromycin at meaningful concentrations.

Using our screening set-up, we identified rifaximin as a clarithromycin potentiator. The combination of rifaximin and clarithromycin was bactericidal *in vitro* and efficacious in a zebrafish embryo infection model. Allthough rifaximin is a non-systemic antibiotic that was approved for treating gastrointestinal bacterial infections, we propose to repurpose rifaximin in combination with clarithromycin for aerosol delivery to treat *M. abscessus* lung infections.

## Materials and methods

### Bacterial strains and culture media

*M. abscessus* strain M422 was used for screening and hit confirmation because it exhibits robust inducible resistance against clarithromycin. For checkerboard synergy assay, three *M. abscessus* strains were used: *M. abscessus* ATCC 19977 and *M. abscessus* strains M422 and M110, whereas bactericidal activity determination and the zebrafish study were performed with *M. abscessus* ATCC 19977. All three strains of *M. abscessus* harbour the inducible clarithromycin resistance-conferring *erm*(41) T28 sequevar. *M. abscessus* strains M422 and M110 were provided by Dr Jeanette Teo, National University Hospital, Singapore.^19^*M. abscessus* ATCC 19977 and *M. avium* ATCC 700898 were acquired from the ATCC.

To visualize the bacterial infection in the zebrafish (*Denio rerio*), a fluorescent strain of *M. abscessus* ATCC 19977 containing pJKD2893:mScarlet was generated. The pJKD2893 plasmid backbone was a kind gift from Professor Timothy Stinear. The gene expressing the fluorescent protein mScarlet was amplified from pMRE135-mScarlet (Addgene #118489, Mitja Remus-Emsermann lab) using the forward primer CTGAGTTCGGCGCCACTAGTTTACTTGTACAGCTCGTCCA and the reverse primer ATTTAAGAAGGAGATATACTATGGTGAGCAAGGGCGAGGC. The amplification product was annealed by Gibson cloning with the pJKD2893 backbone amplified from a sample of pJKD2893 using forward primer AGTATATCTCCTTCTTAAATCTAGAGGATCC and reverse primer ACTAGTGGCGCCGAACT. The resulting plasmid, termed pJKD2893:mScarlet, was electroporated into *M. abscessus* ATCC 19977 at a culture density equivalent to OD_600_ ∼20 using a BioRad GenePulser Xcell set at 2.5 kV, 25 µF and 1000 Ω. After electroporation, the culture was expanded to 2–5 mL and left to recover at 32°C for 24 h in complete 7H9 media without selection drug. The culture was subsequently concentrated to 100 µL and plated on 7H11 supplemented with 100 mg/L kanamycin (GoldBio). Colonies carrying the plasmid were identified by their fluorescence.

All liquid bacterial cultures were grown in Middlebrook 7H9 broth (BD Difco) supplemented with 0.5% albumin, 0.5% glycerol, 0.2% glucose, 0.085% sodium chloride and 0.05% Tween 80. Solid cultures were grown on Middlebrook 7H10 agar (BD Difco) supplemented with 0.5% albumin, 0.5% glycerol, 0.2% dextrose, 0.085% sodium chloride, 0.006% oleic acid and 0.0003% catalase.

### Primary compound screening against preinduced bacterial cells

A total of 2252 drugs/compounds comprising 1576 FDA-approved drugs and 676 compounds in clinical development were purchased from MedChemExpress. The compounds were dissolved in DMSO to a stock concentration of 10 mM. The clarithromycin resistance in *M. abscessus* was induced for 3 days under 0.5 mg/L of clarithromycin (Merck) before being subjected to compound screening. *M. abscessus* strain M422 was selected for the primary screening as it exhibited robust inducible clarithromycin resistance. After 3 days of incubation in an airtight container (Lock and Lock container) with moist paper towels at 37°C, the MIC of clarithromycin increased from 4 mg/L to >64 mg/L. MIC values were determined from dose–response curves using GraphPad Prism version 9 (Dotmatics).

Primary screening was carried out in 96-well flat-bottom cell culture plates (Corning Costar) at 50 µM compound along with 4 mg/L clarithromycin in a starting inoculum of preinduced *M. abscessus* cells at OD_600_ of 0.005 in a final volume of 100 µL. The plates were put in an airtight container with moist paper towels and incubated for 4 days at 30°C. The cultures in the wells were resuspended before OD_600_ was read in a BioTek Synergy 4 plate reader. Compounds were defined as hits if they showed growth inhibition >90% compared with the untreated control.

### Checkerboard synergy assay

With rifaximin identified as the best potentiator of clarithromycin activity, the interaction between rifaximin and clarithromycin was investigated by a checkerboard synergy approach using a broth microdilution method performed in 96-well plates. To provide a classification of the combined antibiotics based on the fractional inhibitory concentration index (FICI), this assay applied the combination of clarithromycin and rifaximin (Merck) in concentrations of 0 to 64 mg/L by 2-fold serial dilutions along the abscissa and ordinate, respectively. The FICI was calculated by summing the FIC of rifaximin and the FIC of clarithromycin. The FIC of clarithromycin is the MIC of clarithromycin in the presence of rifaximin divided by the MIC of clarithromycin alone. The FIC of rifaximin was similarly calculated. The drug interactions between rifaximin and clarithromycin were defined as synergistic when they had a FICI ≤0.5.

### Bactericidal/bacteriostatic activity determination

To determine bactericidal and bacteriostatic activity, a series of MBC assays were performed on *M. abscessus* ATCC 19977. From the checkerboard synergy assay plate, the well with the most synergistic combination of clarithromycin and rifaximin (1  × MIC) was determined. The wells corresponding to the five concentrations (0.5×, 1×, 2×, 4× and 8 × MIC) of rifaximin in the presence of 1 × MIC clarithromycin were plated. Similarly, the wells corresponding to the five concentrations (0.5× , 1× , 2× , 4×  and 8 × MIC) of clarithromycin in the presence of 1 × MIC rifaximin were plated. Serial 10-fold dilutions were performed on the above-mentioned combinations of clarithromycin and rifaximin. Aliquots (50 µL) of each dilution were spread onto 7H10 agar plates, which were incubated at 37°C for 3 days and the cfu counts enumerated. MBC_90_ is defined as the lowest drug concentration required to induce ≥90% cell death compared with the untreated control at 0 h timepoint. The combination of clarithromycin and rifaximin is defined as bactericidal if its MBC_90_ is ≤4×MIC_90_.^20^

### Zebrafish strains, breeding and housing

All work with zebrafish was approved by the Nanyang Technological University Institutional Animal Care and Use Committee (Animal Use Protocol #A20038). Adult zebrafish were housed in a facility kept at 28.5°C and light/dark cycles of 14 h/10 h. Only WT zebrafish of the AB strain were used for these experiments.

### Preparation of M. abscessus inoculum and zebrafish infections

Cultures of *M. abscessus*-pJKD2893:mScarlet in 7H9 media supplemented with 50 mg/L kanamycin were grown to an OD_600_ of 0.2–0.3 and concentrated to an OD_600_ of 1 in 0.1% PBS-Tween-20. The inoculum was prepared by homogenizing the bacterial suspension through a 26 gauge needle and subjecting it to ultrasonication at low power, as described by Bernut *et al*.^21^ The homogenate was left for 10 min for clumps to settle and the supernatant was subsequently moved to a new tube and mixed with phenol red to a final concentration of 0.1%.

Larvae aged 48 h post-fertilization were dechorionated and anaesthetized by immersion with MS-222 (Merck). *M. abscessus*-pJKD2893:mScarlet in 0.1% PBS-Tween-20 and 20% phenol red (2–3 nL containing 300–350 cfu) was injected into the hindbrain ventricle using an Eppendorf FemtoJet microinjector. The actual cfu in the inoculum was determined *a posteriori* by injecting into 50 µL 0.1% PBS-Tween-20 and streaking on a plate. Infected larvae were kept in E3 medium for 24 h, whereupon they were randomly divided into groups for antibiotic treatments. The larvae were treated by immersion in water containing the antibiotics and the water was changed daily.

### Bacterial recovery and cfu determination

Larvae were harvested to assess the bacterial load at 5 days post-infection using a slightly modified version of the Kremer lab method.^21^ Briefly, groups of five larvae were transferred to a 1.5 mL microcentrifuge tube and washed twice with 0.1% PBS-Tween-20. The larvae were homogenized together in 100 µL 0.3% PBS-Triton-X using a hand-held motorized homogenizer with 1.5 mL pestle. The homogenate was run through a 26-gauge syringe, diluted, and spread on 7H11-agar containing 100 mg/L kanamycin, 250 mg/L amphotericin B (Merck) and 25 mg/L hygromycin (Merck). Fluorescent colonies were counted after 5–6 days.

### Statistical analyses

Data were analysed by using two-tailed Student’s *t*-test with Welch’s correction using TTEST function on Microsoft Excel, and a value of *P* < 0.05 was considered statistically significant (*P < 0.05; **P < 0.01). Curve fitting for MIC determinations was achieved using Graphpad Prism (v9, Dotmatic).

## Results

### Identification of rifaximin as a clarithromycin potentiator in M. abscessus

**Figure 1.**
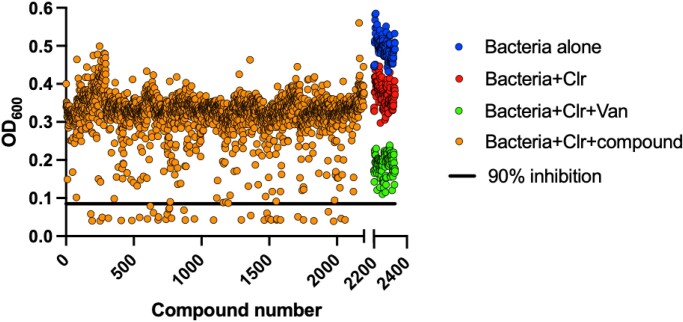
Scatter plot of the screening of 2252 compounds. The compounds that resulted in >90% growth inhibition of *M. abscessus* are defined as hits. The combination of clarithromycin (Clr) and vancomycin (Van) was used as a positive control.

A compound library consisting of 2252 FDA-approved and clinical-stage drugs was screened against *M. abscessus* clinical strain M422. Bacteria were exposed to a subinhibitory concentration of clarithromycin (0.5 mg/L) for 3 days to induce clarithromycin resistance. The MIC of clarithromycin in uninduced *M. abscessus* strain M422 was 4–8 mg/L, a value that shifted to >64 mg/L after exposure to clarithromycin for 3 days (see Figure S1, available as Supplementary data at *JAC-AMR* Online). The test drugs were screened at 50 µM in the presence of 4 mg/L clarithromycin to maintain the clarithromycin-resistance phenotype. Figure 1 shows the scatter plot of the screen.

From the 2252 compounds, only 38 showed >90% growth inhibition. Upon triaging for known antibiotics and performing dose–response curve validation, the 16 confirmed hits (hit rate of 0.7%) included rifaximin, rifabutin and several cephalosporin, oxazolidinone and β-lactam antibiotics. Rifaximin was identified as a promising candidate because it exhibited 100% growth inhibition in the screen. Additionally, the MIC of rifaximin was significantly lowered in the presence of 4 mg/L clarithromycin (see Figure S2).

### The interaction between clarithromycin and rifaximin is synergistic and bactericidal

**Figure 2.**
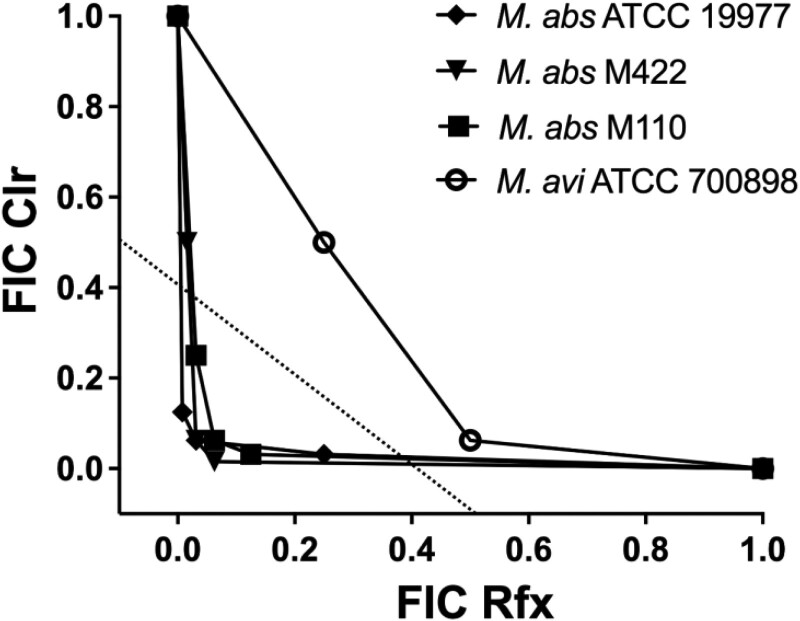
Isobolograms for rifaximin (Rfx) and clarithromycin (Clr) against *M. abscessus* (*M. abs*) and *M. avium* (*M. avi*) strains. The FIC data points that fall below the dotted line (FICI ≤ 0.5) are defined as synergistic. Note that all three strains of *M. abscessus* were preinduced whereas the *M. avium* strain was uninduced.

To characterize the interaction between clarithromycin and rifaximin, checkerboard synergy assays were performed on preinduced clarithromycin-resistant *M. abscessus*. The FICI obtained from the checkerboard assay defines whether the interaction between the two compounds is either synergistic (FICI  ≤0.5), additive (0.5 < FICI ≤ 1.0), indifferent (1.0 < FICI ≤ 4.0) or antagonistic (FICI >4.0).(19) As shown in Table 1 and Figure 2, the combination of clarithromycin and rifaximin exhibited strong synergy (FICI <0.1) in all three *M. abscessus* strains tested. Importantly, the MIC of clarithromycin was reduced by at least 16 times compared with clinically relevant concentrations (2–8 mg/L) when used in in combination with rifaximin (Figure 2, Table 1).

**Table 1.** Checkerboard synergy assay for clarithromycin (Clr) and rifaximin (Rfx) against *M. abscessus* and *M. avium* strains

	MIC_90_ (mg/L)				FICI
*M. abscessus* strain	Individually		In combination		
	Clr	Rfx	Clr	Rfx	
*M. abscessus* ATCC 19977 (preinduced)	128	128	8	4	0.094
*M. abscessus* strain M422 (preinduced)	64	16	2	1	0.094
*M. abscessus* strain M110 (preinduced)	128	128	2	4	0.047
*M. avium* ATCC 700898 (uninduced)	0.5	0.03	0.031	0.015	0.563

Because both *M. abscessus* and *M. avium* complexes are the main organism groups causing pulmonary NTM,^22,23^ we tested the combination of clarithromycin and rifaximin against *M. avium* in the checkerboard assay as well. With a FICI of 0.563, the interaction between clarithromycin and rifaximin was additive in *M. avium*. However, given that clarithromycin and rifaximin are highly potent in *M. avium* (MIC_90_  <0.1 mg/L), the antibiotic combination could still be effective to treat lung infections caused by both *M. abscessus* and *M. avium*.

**Figure 3.**
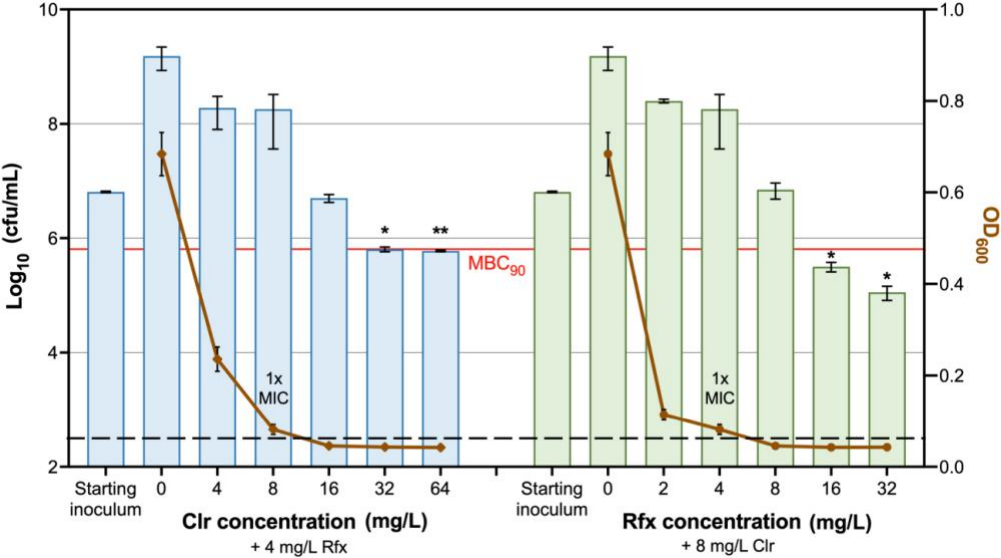
MBC and MIC determination of the combination of clarithromycin (Clr) and rifaximin (Rfx) on *M. abscessus* ATCC 19977. The cfu counts were determined for the cells treated with 0.5×, 1×, 2×, 4× and 8 × MIC of clarithromycin in the presence of 1 × MIC of rifaximin, and vice versa. The MBC_90_ (orange line) was defined as the lowest drug concentration required to induce >90% cell death compared with the starting inoculum (at 0 h timepoint) of the untreated control. The number of residual log_10_ cfu/mL was determined through plating 10-fold serial dilution and compared with the starting inoculum. Statistical significance was determined by a two-tailed Student’s *t*-test with Welch’s correction; *P < 0.05; **P < 0.01. The dashed black line indicates the limit of detection. The experiments were carried out in duplicates; error bars represent the SD.

To determine whether the combination of clarithromycin and rifaximin is bactericidal, *M. abscessus* ATCC 19977 was treated with the antibiotics at 0.5×, 1×, 2×, 4× and 8 × MICs in the presence of 1 × MIC of the partner antibiotic, and bacterial viability was determined by cfu enumeration on agar plates. MBC_90_ was achieved with either clarithromycin 32 mg/L + rifaximin 4 mg/L, or clarithromycin 8 mg/L + rifaximin 16 mg/L (Figure 3), where the criterion of MBC_90_ ≤4 × MIC_90_ is met,^20^ indicating that combining these two antibiotics results in a bactericidal combination.

### The combination clarithromycin and rifaximin is efficacious in a zebrafish embryo infection model

**Figure 4.**
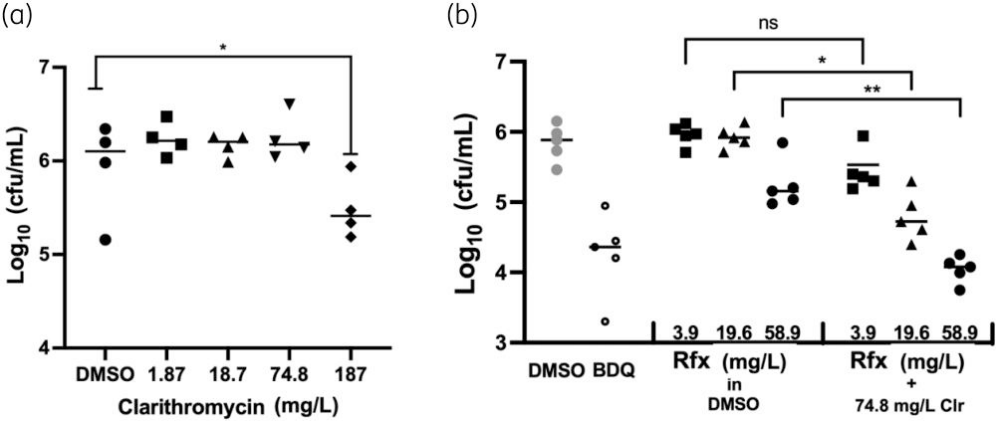
Rifaximin synergizes with clarithromycin to reduce the load of *M. abscessus* in infected zebrafish larvae. (a) Infected fish treated with various concentrations of clarithromycin. (b) The bacterial burdens of animals treated with rifaximin only were compared with those of animals treated with rifaximin and clarithromycin in combination. Bedaquiline (BDQ; 3 mg/L) was used as a control becuase it has demonstrated efficacy against *M. abscessus* in zebrafish embryos.^24^ Note that each dot plotted represents five larvae homogenized together. Fish were infected in the hindbrain ventricle at 48 hours post-fertilization (hpf), and treatment began at 72 hpf. Treatment was maintained for 4 days with daily water changes before the fish were assessed for bacterial burden. Clr, clarithromycin; ns, non-significant; Rfx, rifaximin.

Given the strong synergy observed *in vitro*, the efficacy of the clarithromycin + rifaximin combination was evaluated *in vivo*. The zebrafish embryo infection model was used in this study as it is a well-characterized *in vivo* model system for compound testing against *M. abscessus*.^21^ First, the potency of clarithromycin was evaluated to determine a subinhibitory concentration that could be used in subsequent combination experiments. Infected zebrafish were treated with four different concentrations of clarithromycin ranging from 1.87 mg/L to 187 mg/L (2.5–250 µM). Clarithromycin did not reduce bacterial load up to a concentration of 74.8 mg/L (100 µM) (Figure 4). This concentration was selected as the subinhibitory concentration of clarithromycin to evaluate its interaction with rifaximin in the zebrafish infection model. The combination of 19.6 mg/L (25 µM) rifaximin and 74.8 mg/L clarithromycin resulted in >1 log cfu reduction, whereas 19.6 mg/L rifaximin alone did not show any cfu reduction (Figure 4). Similarly, 58.9 mg/L (75 µM) rifaximin and 74.8 mg/L clarithromycin reduced the bacterial load by >2 log cfu count compared with rifaximin treatment alone, showing that the combination is potent *in vivo* (Figure 4).

## Discussion

NTM treatment routinely involves the use of several antibiotics to enhance efficacy and to minimize the development of resistance. As the cornerstone of *M. abscessus* treatment, clarithromycin is often included in the combination therapies based on anecdotal or clinical experience. The identification of synergistic combinations, however, can be achieved more efficiently by screening combinations of approved drugs *in vitro*, which could accelerate bench-to-bedside translation because the approved drugs have previously been evaluated for safety. Using this approach, we identified rifaximin and showed that it acts synergistically with clarithromycin against *M. abscessus* both *in vitro* and *in vivo*.

Among the rifamycin family, rifabutin was reported in several independent studies to exhibit bactericidal activity against *M. abscessus* and display synergy with clarithromycin.^14,25^ Unsurprisingly then, rifabutin was also identified as one of the top hits in our primary screening. Given that rifaximin and rifabutin are from the same family, rifaximin may inhibit the induction of the *erm(41)* gene by targeting its transcription, thereby enabling clarithromycin to remain effective.^26^

Rifaximin was overlooked in previous *M. abscessus* screening campaigns because *M. abscessus* contains ADP-ribosyltransferase, which accounts for the inactivation and resistance to several rifamycin derivatives including rifaximin.^27,28^ Indeed, we found the MIC of rifaximin is extremely high when tested alone. However, because we set out to specifically search for clarithromycin potentiators by performing compound screening in the presence of clarithromycin, our assay successfully identified this potent combination of rifaximin and clarithromycin. Interestingly, most of the drugs that were previously identified to be synergistic with clarithromycin, such as rifabutin, vancomycin and imipenem, did not maintain the same synergy when tested on preinduced *M. abscessus* (Figure S3).

Rifaximin was approved to treat gastrointestinal infections only because of its low oral bioavailability. Our observation of the synergy between clarithromycin and rifaximin may allow us to leverage the non-systemic nature of rifaximin to treat *M. abscessus* lung infections by administering rifaximin by aerosol delivery. An aerosolized form of rifaximin was used to treat *Pseudomonas aeruginosa* lung infection in mice,^29^ indicating that this approach could be applied to NTM infections.

Pulmonary infection with *M. abscessus* is primarily an extracellular infection of the sputum where the bacteria mainly reside on the epithelial surface of the lung and sputum.^30,31^ It is challenging for an antibiotic administered orally to achieve a high concentration at the interstitial space of lung tissue and sputum in the alveolar lumen.^32,33^ This limitation can be overcome by aerosol administration, which enables drugs of desired concentrations to be loaded directly into the lungs. Furthermore, administering clarithromycin through inhalation could reduce some of the adverse effects compared with the IV route by reducing systemic exposure.^34^ Hence, the proposed approach of nasal co-administration of rifaximin and clarithromycin is a feasible therapeutic option for pulmonary *M. abscessus* infection.

## Supplementary Material

dlad052_Supplementary_Data

## Supplementary data

Figures S1 to S3 are available as Supplementary data at *JAC-AMR* Online.
