# Supplementary material for: Rifaximin potentiates clarithromycin against Mycobacterium abscessus in vitro and in zebrafish
Source: JAC Antimicrob Resist. 2023 May 8;5(3):dlad052. doi: 10.1093/jacamr/dlad052 (PMC10164658; doi:10.1093/jacamr/dlad052)
Supplement: dlad052_Supplementary_Data [file dlad052_supplementary_data.docx]

**Supplemental Materials**

“Rifaximin potentiates clarithromycin against *Mycobacterium abscessus* *in vitro* and in zebrafish” by Boon Chong GOH, Simon Larsson, Linh Chi DAM, Yan Han Sharon LING, Wei Lin Patrina CHUA, Abirami R., Samsher SINGH, Jun Long Ernest ONG, Jeanette W.P. TEO, Peiying HO, Philip W. Ingham, Kevin PETHE, and Peter C. DEDON


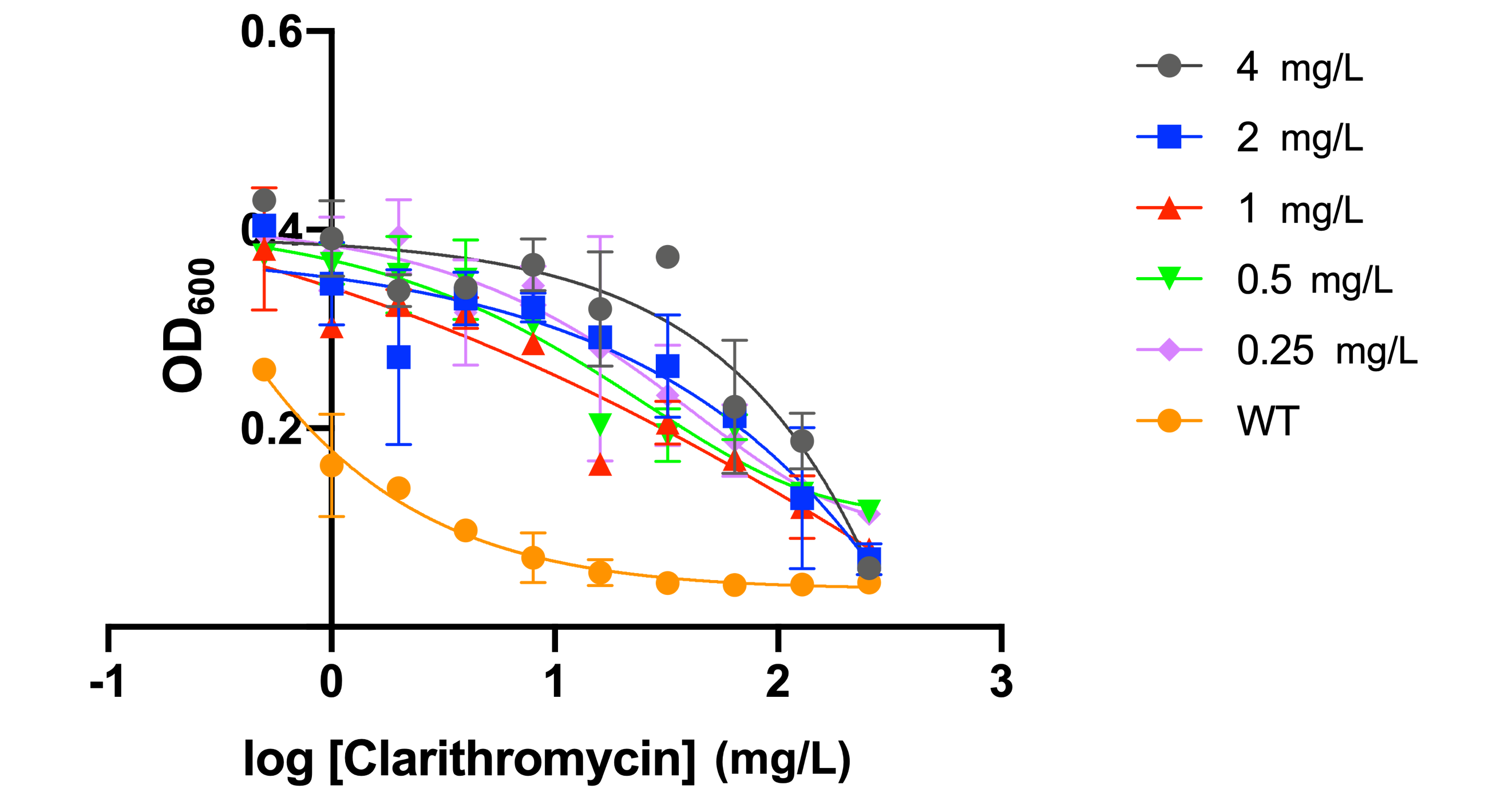


**Figure S1**. MIC of *M. abscessus* strain M422 increases significantly with clarithromycin induction. Cells were grown for 3 days in the presence of clarithromycin at 0.25-4 mg/L to induce resistance, followed by determination of the MIC for clarithromycin. The MIC of uninduced cells was 4-8 mg/L and increased to >64 mg/L following induction of resistance. MIC values were determined from the dose-response curves using GraphPad Prism software (version 9).


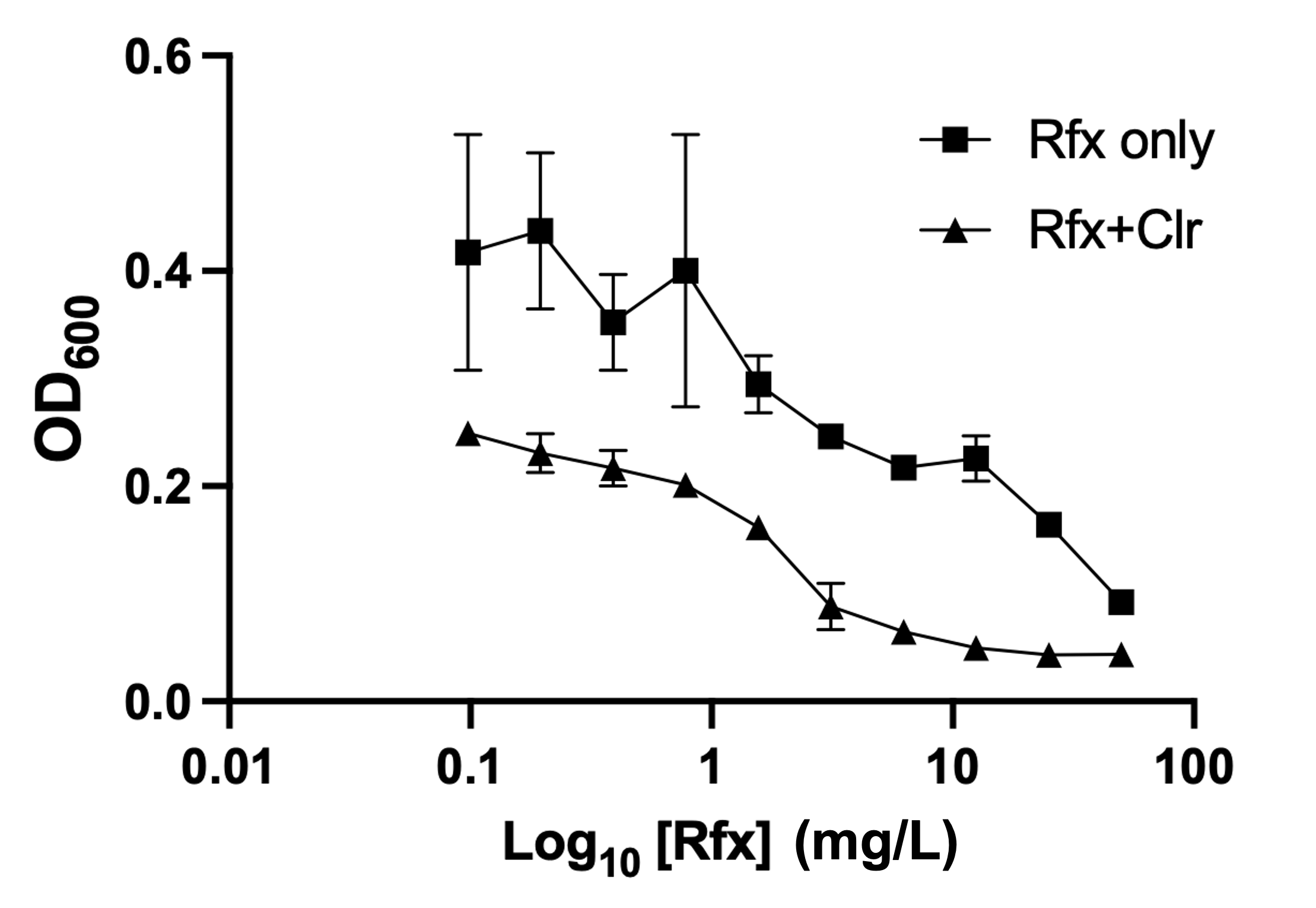


**Figure S2**. Dose response of rifaximin on *M. abscessus* strain M422 with and without 4 mg/L of clarithromycin. The results show that rifaximin inhibits cell growth better in the presence of clarithromycin.

**Figure S3**. Checkerboard synergy assay of clarithromycin with other antibiotics. Cells shaded green indicate growth and below MIC_90_.
